# Development of the Interstitial Cystitis Self-Help and Medical Resources Scale (ICSR) for Women with Interstitial Cystitis

**DOI:** 10.3390/medicina58091183

**Published:** 2022-08-30

**Authors:** Hui-Chun Chen, Lee-Ing Tsao, Chun-Hou Liao, Chieh-Yu Liu

**Affiliations:** 1 School of Nursing, Chang Gung University of Science and Technology, Taoyuan City 333, Taiwan; 2 School of Nursing, National Taipei University of Nursing and Health Sciences, Taipei 112, Taiwan; 3 Department of Urology, Cardinal Tien Hospital, Fu-Jen Catholic University, New Taipei 362, Taiwan; 4 Department of Health Care Management, National Taipei University of Nursing and Health Sciences, Taipei 112, Taiwan

**Keywords:** interstitial cystitis, self-help, medical resources, reliability, validity

## Abstract

*Background and Objectives*: Women with interstitial cystitis (IC) suffer from spontaneous serious bladder pain symptoms without immediate resolution. Women with IC may lack knowledge of how to help themselves. Therefore, a measurement of IC self-help and medical-resource-seeking for women with IC is needed. *Materials and Methods*: This study recruited 100 women with IC from a teaching hospital in Northern Taiwan. The reliability and validity of the Interstitial Cystitis Self-Help and Medical Resources Scale (ICSR) were assessed using expert validity, confirmatory factor analysis (CFA) to test the construct validity, composite reliability to evaluate the internal consistency, and item analysis to test the discrimination validity of each item. *Results*: The results showed that the ICSR had accurate goodness-of-fit indices and the component reliability ranged from 0.42 to 0.83, indicating good reliability and validity. *Conclusions*: The ICSR is recommended for screening the self-help and medical-resource-seeking abilities of women with IC to aid in diagnosing IC and providing more precise medical treatments.

## 1. Introduction

Interstitial cystitis (IC), also called painful bladder syndrome, is a chronic condition that causes bladder pressure or pain; discomfort around the pelvic area; and urinary frequency, urgency, or nocturia in the absence of infection or any other identifiable cause [1]. IC is considered a chronic, spontaneous, and incurable condition [2]. In recent years, increasing studies have emphasized the importance of self-help and seeking medical help for women with IC [3]. To fulfill these needs, the internationalization of disease education and other social resources should be considered by healthcare providers. Previous studies showed that self-help abilities, also known as personal resourcefulness, help to ensure that patients can independently manage their daily activities despite adverse conditions and maintain a good life quality [4,5,6]. Similarly, seeking medical help, similar to social resourcefulness, provides patients with the ability to modify their cognition and behavior to cope with the disease [7,8,9]. Patients with personal resourcefulness are suggested to have self-control skills, including cognitive reframing, problem-solving, delay of gratification, and self-belief [4]. Moreover, seeking social support resources, including professional and non-professional resources, is recommended [7]; however, patients with IC often lack knowledge about disease management and social support [10,11]. A recent systematic review of 34 studies indicated that patients with IC are 4 times more likely to take depressive medications and have a 23% higher risk of suicidal ideation than non-IC patients [12]. However, when patients are able to manage their symptoms, the effects on their mental functioning and psychosocial life can be reduced [3] and their quality of life can be improved.

Patients with IC experience serious pains, which nearly double the number of outpatient visits in this population over the general population [12]. However, most treatments target immediate pain control and their efficacy is limited [12]. Moreover, healthcare providers have limited capability to observe the lifestyle modifications of patients with IC. Patient education and behavior modifications, including stress management, dietary restrictions, and physical activity have become part of the first-line treatment guidelines for pain management [1]. However, previous studies that measure personal or social resourcefulness are limited and focused on the general population or the elderly [4,5,9]. Therefore, this study aimed to develop a new scale that includes concepts of personal resourcefulness and social resourcefulness for patients with IC.

## 2. Materials and Methods

### 2.1. Participants and Setting

This study adopted an exploratory, cross-sectional design and used a convenience sampling method. The study protocol complied with research ethics and was approved by the institutional review board of the teaching hospital in Northern Taiwan (no. CTH-104-3-5-054). From April 2016 to July 2020, outpatients with IC who visited the urology clinic from this teaching hospital were referred by urologists. The inclusion criteria were as follows: women aged 20 to 69 years old; the presence of bladder pressure, bladder pain, or discomfort around the pelvic area in the absence of infection or any other identifiable cause; and cystoscopy data for diagnosing IC. The exclusion criteria were as follows: women with IC who had withdrawn from ketamine therapy and had undergone radiotherapy. The inclusion and exclusion criteria of eligible patients were based on public statistics from the Ministry of Welfare and Health of Taiwan [13]. After obtaining written consent, in-depth, face-to-face interviews were conducted in an independent room at the teaching hospital. Each eligible participant completed the IC Self-Help and Medical Resources Scale (ICSR) survey in 30 to 60 min.

The sample size was calculated based on a study by Anderson and Gerbing [14], who proposed a sample size of at least 100 cases to construct structural equation models or utilize confirmatory factor analysis (CFA).

### 2.2. Instrument Development

**Figure 1:**
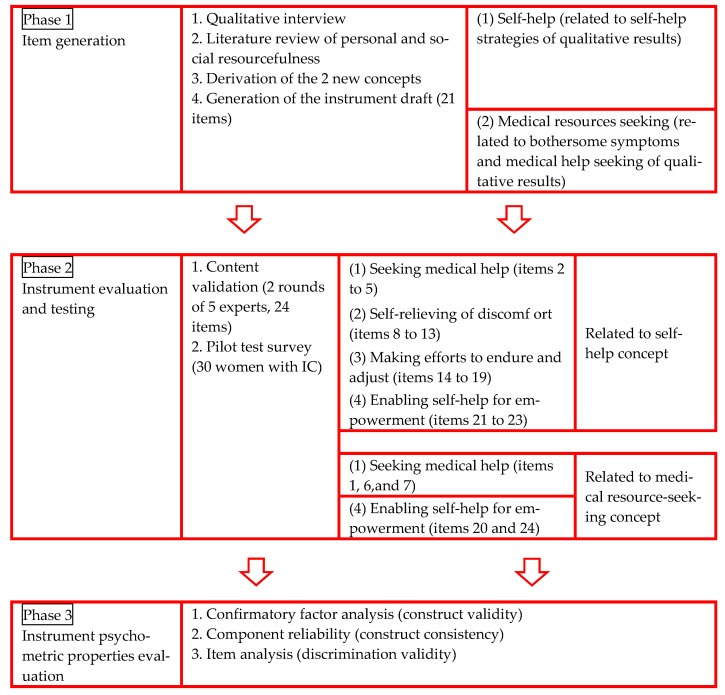
Flow chart of the development of the ICSR.

The ICSR combined personal and social resourcefulness concepts and followed the conceptual framework based on the first stage of qualitative results from women with IC [3] to derive the new concepts: self-help and medical resources. The flow chart of the development of the ICSR is shown in Figure 1.

Phase 1—item generation: the 4 processes of the generation of the items were as follows:

1. Qualitative interview: We assessed the self-perception of symptoms, medical-help-seeking behaviors, and self-help strategies for women with IC. We recruited 68 women with IC, 98.5% of whom did not have ulcerative IC (Hunner’s lesions), and about 72.1% of the patients indicated that their symptoms interfered with their daily activities. The interview guide was developed based on a literature review of resourcefulness concepts, and a 1-to-1 interview format was adopted to collect the data. Three important themes were identified in the interviews: (1) bothersome symptoms—all-day bladder pain, lower urinary tract symptoms, and deteriorated quality of life; (2) medical-help-seeking—exhaustion and frustration; and (3) self-help strategies—coexisting with IC or feeling helpless [3].
2. Literature review of the concepts: findings from a literature review led to the proposal of 2 concepts, namely, personal resourcefulness and social resourcefulness.
3. Derivation of 2 new concepts: Based on the qualitative inquiry results and the literature review, we derived 2 new concepts: self-help, which indicated the ability to adapt self-control skills to cope with their disease, and seeking medical resources, which illustrated the ability to use medical resources to cope with the disease. The bothersome symptoms and medical-help-seeking themes were related to the concept of medical resources seeking, and the self-help strategies theme was related to the concept of self-help.
4. Generation of the instrument draft: the first version of the scale was drafted, which comprised 21 questions, including 19 closed questions and 2 open questions.

Phase 2—instrument evaluation and testing: the 2 processes of instrument evaluation and testing were as follows:

1. Content validation: Two rounds of 5 expert panels were conducted, and as a result, 3 statements were added. These statements were (1) “when I have urinary discomfort, I will seek medical help immediately”; (2) “when I have urinary discomfort, I will seek complementary and alternative medicine immediately, including folk medicine, pray to the gods, and seek to ascertain by divination”; and (3) “when I feel urinary discomfort and pain, I try to take dietary supplementation suggestions from others or by searching the internet, including dried longan, red dates, or ginger tea.” Therefore, after 2 rounds of expert panels, the revised version of the ICSR included 24 questions: 22 closed questions and 2 open questions. The 4 constructs and their items were defined as follows: (1) seeking medical help (items 1 to 7), (2) self-relieving of discomfort (items 8 to 13), (3) making efforts to endure and adjust (items 14 to 19), and (4) enabling self-help for empowerment (items 20 to 24). The 4 constructs of the ICSR related to the second phase and the 2 newly derived concepts of our study were as follows: (1) seeking medical help (items 2 to 5), self-relieving of discomfort (items 8 to 13), making efforts to endure and adjust (items 14 to 19), and enabling self-help for empowerment (items 21 to 23) were related to the concept of self-help; (2) seeking medical help (items 1, 6, and 7) and enabling self-help for empowerment (items 20 and 24) were related to the concept of medical-resource-seeking. Experts used the item-level content validity index (I-CVI) to judge each item as relevant or clear, the scale-level content validity index (S-CVI) to judge each construct as relevant or clear, and the S-CVI average (S-CVI/Ave) to judge the overall scale as relevant or clear [15,16,17]. The I-CVI, S-CVI, and S-CVI/Ave were all 1.00, which illustrated that the overall scale was relevant and clear.
2. Pilot test survey: A pilot study of 30 women with IC was conducted to evaluate the participants’ understanding of each item. Overall, the women indicated that each item was clear [15].

Phase 3—psychometric properties instrument: the 3 processes of the evaluation of the psychometric properties of the instrument were as follows:

1. CFA was used to examine how the item scores from the participants’ responses resulted in goodness-of-fit measures of the factor structure, which indicated good construct validity [18].
2. Component reliability (CR) was used to assess the internal consistency of the participants’ responses for each construct, which was measured using Cronbach’s α, so that unreliable items could be adjusted or removed if necessary [18].
3. Comparisons of extreme groups of item analysis were used to assess the differences between the participants in the high-scoring group and the low-scoring group, which indicated good discrimination validity [18].

### 2.3. Data Analysis

Categorical variables are displayed as the number of cases (n) and percentage (%), while continuous variables are displayed as the mean and standard deviation (SD). Statistical analysis was conducted using SPSS version 20.0 (SPSS Inc., Chicago, IL, USA). CFA and CR were conducted using LISREL version 8.8, Lincolnwood, IL: Scientific Software International, Inc, USA. A 2-tailed *p*-value < 0.05 was considered statistically significant.

## 3. Results

### 3.1. Demographics

A total of 100 women with IC were recruited. The mean age was 55.56 ± 13.92 years (range, 21–73), and the period of self-perceived bothersome IC ranged from 0.5 to 10 years, with a mean period of 3.24 ± 3.19 years. Additionally, 80.0% of the patients were married, 53.0% had not graduated high school, 49.0% had no religion, and 51.0% had no occupation (Table 1). The finalized version of the ICSR is provided in Appendix A.

### 3.2. Validity and Reliability

#### 3.2.1. Content Validity

The ICSR consisted of 24 items, with 4 constructs defined as follows: (1) seeking medical help (items 1 to 7), (2) self-relieving of discomfort (items 8 to 13), (3) making efforts to endure and adjust (items 14 to 19), and (4) enabling self-help for empowerment (items 20 to 24). Moreover, seeking medical help (items 2 to 5), self-relieving of discomfort (items 8 to 13), making efforts to endure and adjust (items 14 to 19), and enabling self-help for empowerment (items 21 to 23) were related to the concept of self-help, while seeking medical help (items 1, 6, and 7) and enabling self-help for empowerment (items 20 and 24) were related to the concept of medical resource-seeking. After the two rounds of expert panels, the experts gave an overall rating of the items of the ICSR as highly relevant or clear, with I-CVI, S-CVI, and S-CVI/Ave values of 1.0.

#### 3.2.2. Construct Validity

This study used CFA to test the construct validity of the ICSR and evaluate the offending estimate and goodness-of-fit indices. The offending estimate results of the 22 items (excluding the two open questions) were as follows: (1) the standard error ranged from 0.05 to 0.09 with no negative standard error; (2) the absolute value of the standardized coefficient (lambda-X) ranged from 0.01 to 0.93; and (3) the standard error of the indicator (delta) range from 0.03 to 0.62 with no negative standard error, which was within the acceptable-to-well range of the estimation parameter, suggesting that the results of the model estimate had a well goodness-of-fit [19].

The first-time goodness-of-fit of the overall model of the ICSR was shown in the RMSEA (root-mean-square error of approximation) to be 0.14, which was higher than the normal range of ≤0.05 [19]. Additionally, the GFI (goodness-of-fit index) was 0.64, and the AGFI (adjusted goodness-of-fit index) was 0.56, which were both below the normal range of values ≥ 0.90 [19]. The factor loadings of 3 of the 22 items ranged from −0.08 to 0.10, which was lower than the acceptable score defined as >0.30 and were considered for deletion [19]; these three items were as follows: (1) “when I have urinary discomfort, I will seek medical help immediately (item 6)”; (2) “when I have urinary discomfort, I will seek complementary and alternative medicine immediately, including folk medicine, pray to the gods and seek to ascertain by divination (item 7)”; and (3) “when I feel urinary discomfort and pain, I try to take dietary supplementation suggestions from others or by searching the internet, including dried longan, red dates, or ginger tea (item 24).” In addition, the modification index value of 2 of the 22 items was 27.33, which was higher than the acceptable value of <3.84, and were considered to be mergedm [19]; the two items were as follows: (1) “when I going out, I choose to go to the restroom conveniently in order to cope with my frenquent urinary discomfort (item 15)” and (2) “when I travel a long time, I choose to take the useful utility of toilet, including take the metro, train, or high speed rail (item 16).” The revised version of the first-time goodness-of-fit indices of the model included 19 items, with four constructs in two concepts. Seeking medical help (items 2 to 5), self-relieving of discomfort (items 8 to 11, and two open questions not calculated (items 12 and 13)), making efforts to endure and adjust (items 14 to 19), and enabling self-help for empowerment (items 21 to 23) were related to the concept of self-help. Seeking medical help (item 1) and enabling self-help for empowerment (item 20) were related to the concept of medical resource-seeking.

**Figure 2:**
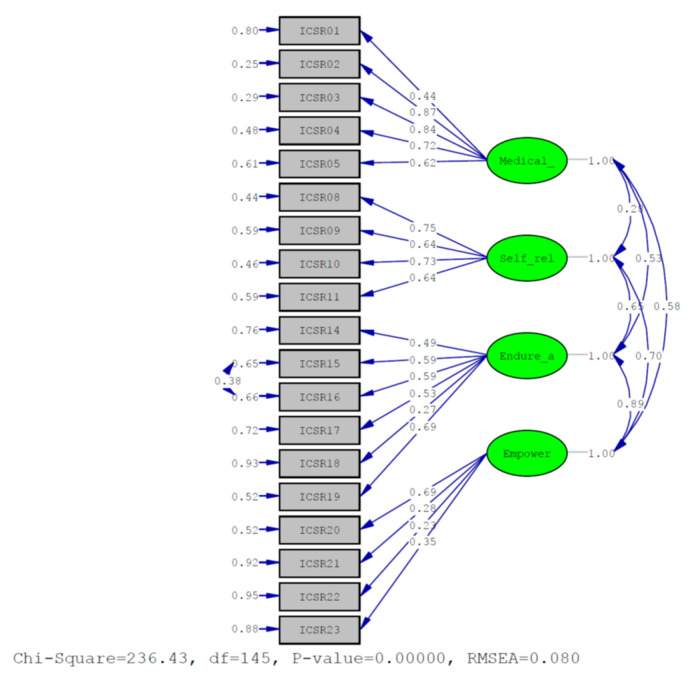
Four-factor model of the ICSR. Notes: Medical: seeking medical help; Self_rel: self-relieving of discomforts; Endure_a: making efforts to endure and adjust; Empower: enabling self-help for empowerment.

After deleting the low goodness-of-fit items and conducting the CFA again, the four-factor model indicated good goodness-of-fit indices (RMSEA = 0.080; PNFI = 0.83; SRMR = 0.094; GFI = 0.93; AGFI = 0.91; PGFI = 0.71) (Table 2). The estimated RMSEA was< 0.05, the PNFI and PGFI were≥ 0.5, the SRMR ranged from 0 to 1, and the GFI and AGFI were ≥0.9 [19]. These values indicated that the best model that was adapted in this study was fit for research. The standardized factor loading model ranged from 0.23 to 0.87. The loading of three items (items 18, 21, and 22) ranged from 0.23 to 0.28, which was lower than 0.3; however, to stay consistent with the qualitative inquiry results of phase 1, items 18, 21, and 22 were retained in the scale (Figure 2). In summary, the four-factor scale with 19 items was finalized (Appendix A).

#### 3.2.3. Construct Reliability

We summarized the factor loading of each item of each construct, squared the factor loading, divided the factor loading of each item of each construct, and then squared the factor loading and summed the residue of each item of each construct to calculate the CR of the CFA [19]. Table 3 shows the CR of each construct. A CR value of ≥0.7 indicated good internal consistency [19]. These results indicated that the construct of enabling self-help for empowerment did not have good internal consistency, suggesting that responses regarding this construct were inconsistent. 

#### 3.2.4. Discrimination Validity

The results of the item analysis indicated that 18 of the 19 items exhibited statistically significant differences between the high-score group and the low-score group (upper 27% and lower 27%). Specifically, the *t*-values of the 18 items ranged from 2.521 to 6.899, which indicated that these items showed statistically significant differences and had good discrimination validity [18]. However, 1 (item 21) of the 19 items did not differ significantly between the high- and low-scoring groups, suggesting that all of the participants responded to the item consistently [18] (Table 4).

## 4. Discussion

This 19-question ICSR was developed and validated as a new, self-reported instrument to measure the self-help and medical resources for women with IC. In this study, we derived two new concepts from our qualitative inquiry results [3] and performed a literature review of two concepts of resourcefulness [4,7]. Self-help was one concept of the ICSR, which consisted of four constructs, namely, seeking medical help, self-relieving of discomfort, making efforts to endure and adjust, and enabling self-help for empowerment, which differed from the findings of Rosenbaum [4], who reported the constructs of the ability of cognitive reframing, problem-solving, delay of gratification, and self-belief. This difference may have been because, in the current study, participants were dedicated to performing specific tasks to cope with their discomfort. Therefore, fewer women were willing to report weaker coping with their disease, and general daily tasks need to be explored further. Medical resources made up the other concept of the ICSR, which comprised seeking medical help and enabling self-help for empowerment as constructs. This result was consistent with a report by Nadler [7], who reported on the ability to seek professional and non-professional advice on adaptive behavior. 

This study used CFA to examine the construct validity. The dimension structure of ICSR was based on the results of a qualitative interview, and the reliability and validity were investigated using different goodness-of-fit indices. This study used two rounds of CFA to examine the goodness-of-fit indices. The first round resulted in deleting three items because the factor loading was <0.3 [19], implying that these items were less related to their construct. This finding differed from that of a study by Chen et al. [3], which reported that participants sought medical help after suffering a urinary problem and tried to take more mild dietary supplements for self-help. The first round of CFA also suggested combining two items for which the modification index was <3.84 [19] since these items tested similar ideas. These results were similar to those of the study by Chen et al. [3], which reported that participants did not hold their urine in for a long time to relieve their urinary problems. The final version of the ICSR consisted of 19 items with four factors. The results of the CFA model were similar to the results of the qualitative interviews, which suggested that the construct of the ICSR conformed to the initial concepts of the qualitative inquiry. 

In terms of CR, three of the four constructs had acceptable CR values of 0.75 to 0.83, while one was below the acceptable value [19], which suggested inconsistent responses regarding this construct. This finding differs from that of the study by Malde et al. [1], which reported that timed voiding as an adaptive behavior, dietary manipulation, and stress reduction were the first-line treatment guidelines given to educate patients. These differences suggest inconsistencies in the points of view of healthcare providers regarding educational resources and of patients regarding useful resources, perhaps due to cultural differences. Taiwanese women often comply with the suggestions of healthcare providers and do not often express difficult execution of daily activities. After considering the cultural characteristics of Taiwanese women, this construct was retained in the ICSR. 

The ICSR demonstrated that 18 of the 19 items had good discrimination validity, as the high- and low-scoring participant groups had statistically significant differences [18]. The remaining item, “I will try to not hold my urine for a long time when I have a lot of work to do and need the toilet badly (item 21)”, did not differ significantly between the groups, suggesting that most of the participants did not hold their urine for a long time. This finding differs from that of the study by Malde et al. [1], which reported that patients were educated to use timed voiding as adaptive behavior. This difference may be because participants often held their urine before receiving an IC diagnosis, and will not hold urine for a long time due to a fear of the disease relapsing. Therefore, fewer participants tried timed voiding for bladder training, and most participants will not hold their urine for a long time. Overall, the ICSR had acceptable discrimination validity, and this item was retained in the final ICSR.

This study had some limitations. First, the age of the participants in this study ranged from 20 to 69 years. Therefore, the findings of this study cannot be generalized to women that are more than 69 years old. Second, the sample size was only 100, and participants were recruited from one teaching hospital in Northern Taiwan. However, Anderson and Gerbing [14] indicated that a sample size of 100 meets the minimum requirement for evaluating structural equation models or using CFA. 

## 5. Conclusions

In conclusion, the newly developed ICSR had good validity and reliability and can be recommended to urologists and nurses to evaluate the self-help and medical-resource-seeking abilities of women with IC. With the ICSR, healthcare providers can provide more comprehensive healthcare for women with IC. 

## Figures and Tables

**Table 1:** Participant characteristics (*N* = 100).

Variable	*n* (%)	Median (Min ^1^–Max ^2^)	Mean ± SD ^3^
Age (years)		61.50 (21.00–73.00)	55.56 ± 13.92
Self-perceived bothersome IC ^4^ symptoms (years)		2.00 (0.50–10.00)	3.24 ± 3.19
Married status			
Unmarried	11 (11.0)		
Married	80 (80.0)		
Divorced and widowed	9 (9.0)		
Education			
Under senior high school	53 (53.0)		
Associate degree	16 (16.0)		
College or university or above	31 (31.0)		
Religion			
None	49 (49.0)		
Buddhism	33 (33.0)		
Taoism	9 (9.0)		
Christianity or Catholicism	9 (9.0)		
Occupation			
Yes	49 (49.0)		
No	51 (51.0)		

^1^ Abbreviation: Min, minimum. ^2^ Abbreviation: Max, maximum. ^3^ Abbreviation: SD, standard deviation. ^4^ Abbreviation: IC, interstitial cystitis.

**Table 2:** Construct validity of the ICSR.

Four-Factor Model	RMSEA ^1^	PNFI ^2^	SRMR ^3^	GFI ^4^	AGFI ^5^	PGFI ^6^
Score	0.080	0.83	0.094	0.93	0.91	0.71

^1^ RMSEA—root-mean-square error of approximation; ^2^ PNFI—parsimony normed fit index; ^3^ SRMR—standardized root-mean-square residual; ^4^ GFI—goodness-of-fit index; ^5^ AGFI—adjusted goodness-of-fit index; ^6^ PGFI—parisimony goodness-of-fit index.

**Table 3:** Construct reliability of the ICSR.

Construct of ICSR	Number of Items	Component Reliability
Seeking medical help	5	0.83
Self-relieving of discomfort	4	0.79
Making efforts to endure and adjust	6	0.75
Enabling self-help for empowerment	4	0.42

**Table 4:** Comparisons of the high- and low-scoring groups in the ICSR (*N* = 100).

Items	*t*-Value	*p*-Value
1. If I do not feel better regarding my urinary problems, I will transfer to another hospital or physician for examination.	2.943	0.005
2. I worry about drug addiction after taking medicine.	5.877	<0.001
3. I worry about adverse effects after taking medicine.	6.009	<0.001
4. I am afraid that when I discontinue my medication, I will experience recurrence of my urinary problem.	4.501	<0.001
5. I feel inconvenienced by taking medicine when I go out, and I feel it disturbs my emotions.	5.039	<0.001
8. I found that when I pay attention to a task, the feeling of pain or discomfort is relieved.	6.899	<0.001
9. I found that when I am doing some activities, including walking, jogging, riding a bicycle, or yoga, the feeling of pain or discomfort is relieved.	5.049	<0.001
10. I found that when I message my abdomen, the feeling of pain or discomfort is relieved.	4.915	<0.001
11. I found that when I lie in bed, the feeling of pain or discomfort is relieved.	3.921	<0.001
14. I will choose a job that offers a replacement or substitute, including packer, restaurant server, office work, or store staff, to cope with my frequent urination.	4.948	<0.001
15. when I go out, I choose to go to the restroom conveniently to cope with my frequent urinary discomfort.	5.007	<0.001
16. when I travel a long time, I choose to take the useful utility of toilet, including take the metro, train, high speed rail	5.975	<0.001
17. I can tolerate diapers or sanitary napkins to cope with my frequent urinary discomfort.	4.633	<0.001
18. I will drink more water or soup in the daytime to cope with my urinary problem.	2.521	0.015
19. I will endure the inconvenience of going to the restroom frequently to cope with my frequent urination.	6.488	<0.001
20. I will follow the advise of healthcare providers to resolve my urinary problems.	6.104	<0.001
21. I will try to not hold my urine for a long time while I have a lot of work to do and I need the toilet badly.	1.347	0.189
22. I will not eat food that can stimulate the bladder, including caffeine, tea, alcohol, chocolate, spicy, or cranberry, to cope with urinary discomfort.	2.759	0.008
23. I will try to do something for relief or to make me happy, including watch a movie, take a bath, sign a song, or listen to music, when I feel pain or discomfort from my urinary problem.	4.735	<0.001

## Data Availability

Not applicable.

## Appendix A

**Table A1:** IC Self-Help and Medical Resources Scale (ICSR).

**Constructs and Items**	**Strongly Disagreed**	**Disagreed**	**Agreed**	**Strongly Agreed**
**A.** **Seeking medical help**				
**1. If I do not feel better regarding my urinary problems, I will transfer to another hospital or physician for examination.**	□	□	□	□
**2. I worry about drug addiction after taking medicine.**	□	□	□	□
**3.** **I worry about adverse effects after taking medicine.**	□	□	□	□
**4. I am afraid that when I discontinue my medicine, I will experience** **recurrence of my urinary problem.**	□	□	□	□
**5. I feel inconvenienced by taking medicine when I go out, and I feel it disturbs my emotions.**	□	□	□	□
**B. Self-relieving of discomforts**				
**6. I found that when I pay attention to a task, the feeling of pain or discomfort is relieved.**	□	□	□	□
**7. I found that when I am doing some activities, including walking, jogging, riding a bicycle, or yoga, the feeling of pain or discomfort is relieved.**	□	□	□	□
**8. I found that when I message my abdomen, the feeling of pain or discomfort is relieved.**	□	□	□	□
**9. I found that when I lie in bed, the feeling of pain or discomfort is relieved.**	□	□	□	□
**C. Making efforts to endure and adjust**				
**10. I will choose a job that offers a replacement or** **substitute, including packer, restaurant server, office work, or store staff, to cope with my frequent urination.**	□	□	□	□
**11. When I go out, I choose to go to the restroom conveniently to cope with my frequent urinary discomfort.**	□	□	□	□
**12. when I travel a long time, I choose to take the useful utility of toilet, including take the metro, train, high speed rail**	□	□	□	□
**13. I can tolerate diapers or sanitary napkins to cope with my frequent urination discomfort.**	□	□	□	□
**14. I will drink more water or soup in the daytime to cope with my urinary problem.**	□	□	□	□
**15. I will endure the inconvenience of going to the restroom frequently to cope with my frequent urination.**	□	□	□	□
**D. Enabling self-help for empowerment**				
**16. I will follow the advice of healthcare providers to resolve my urinary problems.**	□	□	□	□
**17. I will try to not hold my urine for a long time when I have a lot of work to do and need the toilet badly.**	□	□	□	□
**18. I will not eat food that can stimulate the bladder, including caffeine, tea, alcohol, chocolate, spice, or cranberry, to cope with urinary discomfort.**	□	□	□	□
**19. I will try to do something for relief or to make me happy, including watch a movie, take a bath, sign a song, or listen to music, when I feel pain or discomfort from my urinary problem.**	□	□	□	□
